# Overexpressing Ribosomal Protein L16D Affects Leaf Development but Confers Pathogen Resistance in Arabidopsis

**DOI:** 10.3390/ijms24119479

**Published:** 2023-05-30

**Authors:** Ke Li, Zhenwei Yan, Qian Mu, Qingtian Zhang, Huiping Liu, Fengxia Wang, Ao Li, Tingting Ding, Hongjun Zhao, Pengfei Wang

**Affiliations:** 1 Shandong Academy of Grape, Shandong Academy of Agricultural Sciences, Jinan 250100, China; like_sdu@foxmail.com (K.L.); muqian1234aabb@163.com (Q.M.); tcszqt@163.com (Q.Z.); 17862973825@163.com (H.L.); wangfengxia1001@163.com (F.W.); liao429@126.com (A.L.); rainbowisting@163.com (T.D.); 13964190239@139.com (H.Z.); 2 Maize Research Institute, Shandong Academy of Agricultural Sciences, Jinan 250100, China; yanzwplant@sina.com; 3 Key Laboratory of East China Urban Agriculture, Ministry of Agriculture and Rural Affairs, Shandong Academy of Agricultural Sciences, Jinan 250100, China

**Keywords:** ribosomal protein, RPL16D, curled leaf, smaller cell size, immune response

## Abstract

In plant cells, multiple paralogs from ribosomal protein (RP) families are always synchronously expressed, which is likely contributing to ribosome heterogeneity or functional specialization. However, previous studies have shown that most RP mutants share common phenotypes. Consequently, it is difficult to distinguish whether the phenotypes of the mutants have resulted from the loss of specific genes or a global ribosome deficiency. Here, to investigate the role of a specific *RP* gene, we employed a gene overexpression strategy. We found that Arabidopsis lines overexpressing *RPL16D* (*L16D-OEs*) display short and curled rosette leaves. Microscopic observations reveal that both the cell size and cell arrangement are affected in *L16D-OEs*. The severity of the defect is positively correlated with RPL16D dosage. By combining transcriptomic and proteomic profiling, we found that overexpressing *RPL16D* decreases the expression of genes involved in plant growth, but increases the expression of genes involved in immune response. Overall, our results suggest that RPL16D is involved in the balance between plant growth and immune response.

## 1. Introduction

Ribosomes are the ribonucleoprotein complexes that are responsible for protein translation in cells. Like DNA replication and transcription, protein translation is also a basic component of the central dogma of molecular biology. In eukaryotes, the cytosolic ribosome comprises 60S large and 40S small subunits. The 60S large subunit is composed of 25/28S, 5.8S, and 5S rRNA and about 47 RPs (RPLs), while the 40S small subunit is composed of 18S rRNA and approximately 33 RPs (RPSs) [1,2]. In Arabidopsis, each of the 80 *RP* families contains between two and seven paralogs that share 65–100% protein sequence identity [3,4,5]. Furthermore, the paralogs of many RP families are always synchronously expressed and incorporated into maturing ribosomes [3,4]. In contrast, only one gene from an *RP* family is expressed in mammals, and one or two genes from an *RP* family are expressed in yeast [6,7]. In Arabidopsis, duplicated genes within an *RP* family are essential for maintaining the threshold dosage of an RP and always have non-redundant functions [8,9,10,11]. Importantly, studies have shown that some RPs have specialized functions, for example, RPL5A, RPL9C, RPL10aB, and RPL28A in leaf polarity development and RPL10A and RPL24B in the translation of certain mRNAs [9,12].

In Arabidopsis, *RPs* in each family are also specialized by their variation in expression patterns and/or protein dosages. For example, the *RPS5* family is encoded by *RPS5A* and *RPS5B*. Promoter-driven GUS experiments showed that *RPS5A* is enriched in meristem regions, while *RPS5B* is restricted to differentiating tissues [13]. In the *RPL16* family, *RPL16A* is expressed in the root stele and anthers, while *RPL16B* is enriched in proliferating tissues [14]. These studies suggest that different RP paralogs may have different biological relevance and lead to specialized ribosomes in different tissues. However, *RP* mutants normally display similar developmental phenotypes or lack obvious morphological phenotypes [2,4,15]. For example, aberrant vasculature, narrow and pointed leaves, retarded root growth, and abnormal embryogenesis are frequently observed in *RP* mutants [8,9,12,13,15,16,17,18,19,20,21,22]. Lacking specific phenotypes from *RP* mutants hinders the research of specific RPs and ribosome heterogeneity.

In this study, we investigate the functions of specific RPs and their ribosomes by overexpressing specific *RPs* under the control of the CaMV *35S* promoter. We found that increasing the RPL16D protein inhibited plant growth while protecting the plant from pathogen attack. This study provides a new insight into the function of specific RPs and the importance of ribosome heterogeneity in plants.

## 2. Results

### 2.1. Overexpression of RPL16D Displays Short and Curled Rosette Leaves

**Figure 1:**
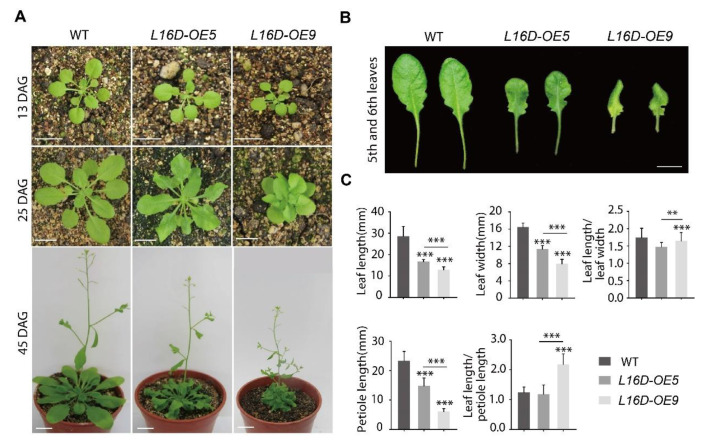
Overexpressing *RPL16D* leads to a curly leaf phenotype. (**A**) The phenotypes of 13, 25, and 45 DAG plants of wild-type (WT), *L16-OE5*, and *L16-OE9*. Scale bars, 15 mm; and (**B**) the fully expanded fifth and sixth rosette leaves (25 DAG). Scale bars, 8 mm. (**C**) Quantitative measurements of the leaf length and width, leaf length/width ratio, petiole length, and leaf length/petiole length ratio of the fifth leaf at 25 DAG. The values shown are averages ± standard errors (n = 15). (** *p* < 0.01 and *** *p* < 0.001, Student’s *t*-test).

To study the function of specific *RP* genes in Arabidopsis, we created different *RPs-GFP* fusion sequences which were driven by the *35S* promoter (*35S:RPs-GFP*) and introduced them into WT plants. The GFP tag was used to compare the expression levels of *RP-GFP* among different lines. In this study, Arabidopsis lines overexpressing *RPL16D-GFP* (*L16D-OE*) were further investigated, and two representative lines (*OE5* and *OE9*) were used to identify the phenotype. No obvious phenotypic difference was observed between *L16D-OE*s and WT before 20 DAG (days after germination) (Figure 1A). However, *L16D-OE*s displayed a striking feature of short and curled leaves after 20 DAG (Figure 1A). This phenotype became more dramatic at later developmental stages (such as 45 DAG), especially in the *L16D-OE9* line (Figure 1A). The petioles of the *L16D-OE9* leaves were short, leading to a bunched-rosette phenotype (Figure 1A). The fifth and sixth leaves of the *L16D-OE5* and *L16D-OE9* lines displayed an abnormal curvature and could not be flattened, which were different from the WT leaves with flattened surfaces (Figure 1B). The leaf length and width and petiole length of *L16D-OE5* and *L16D-OE9* lines were significantly shorter than those of WT (Figure 1C). These phenotypes were more obvious in *L16D-OE9* line than those in the *L16D-OE5* line (Figure 1C). The leaf length/width ratio was not correlated (Figure 1C). We also obtained lines overexpressed in *RPL16D* without a GFP tag (*35S:RPL16D*). The *35S:RPL16D* lines displayed similarly short and curled rosette leaves, ruling out the possibility that the chimeric protein of RPL16D-GFP played a dominant-negative role by competing with the native RPL16D (Figure S1).

**Figure 2:**
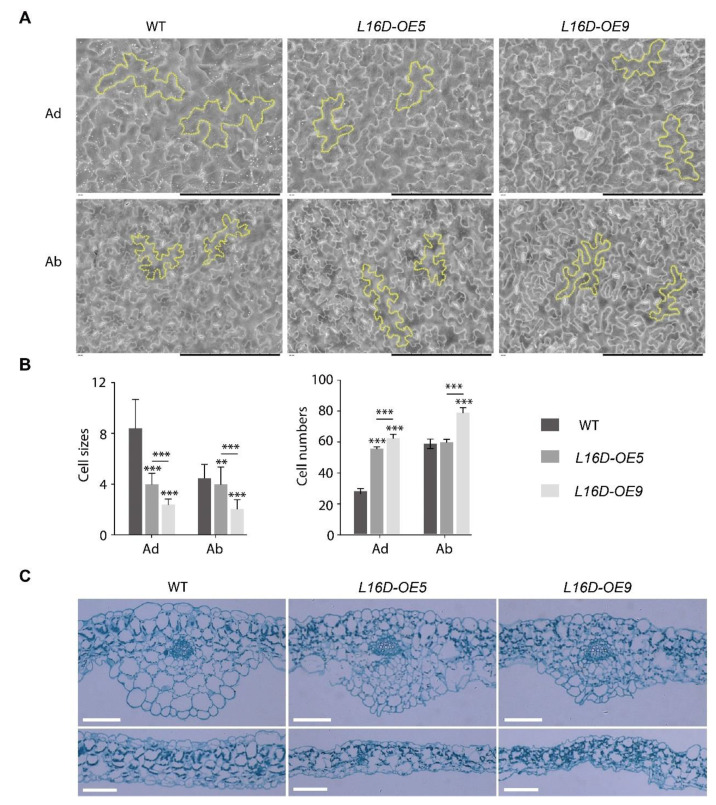
Pavement cell analysis of *L16-OEs* lines. (**A**) Scanning electron micrographs on the adaxial (Ad) and abaxial (Ab) surfaces of the fifth leaf of WT, *L16-OE5*, and *L16-OE9* at 21 DAG. Yellow outlines indicated the representative cells. Scale bars, 200 μm; (**B**) quantitative measurement of cell size and cell number. Values shown are averages ± standard errors (n ≥ 10). (** *p* < 0.01 and *** *p* < 0.001, Student’s *t*-test); and (**C**) cross-sections of the fifth leaf. Scale bars, 100 μm.

The epidermal pavement cells from the fully expanded fifth leaf of WT, *L16D-OE5*, and *L16D-OE9* plants were analyzed by scanning electron micrography (SEM) on the adaxial (Ad) and the abaxial (Ab) surfaces (Figure 2A). Both *L16D-OE5* and *L16D-OE9* showed a reduced epidermal cell size and an increased cell number compared to those in WT, indicating that the reduced leaf size of *L16D-OEs* plants might be due to the reduced cell expansion (Figure 2A,B). The leaf cross-sections of *L16D-OE5* and *L16D-OE9* showed abnormal cell arrangement and changed cell sizes in both the Ad and Ab epidermis (Figure 2C). The structure of the upper leaf mesophyll cell and lower leaf palisade tissue was also abnormal in both the arrangement and cell shape (Figure 2C). Taken together, our results indicate that a short and curled leaf morphology in *L16D-OEs* was associated with a reduced cell size and disordered cell growth.

**Figure 3:**
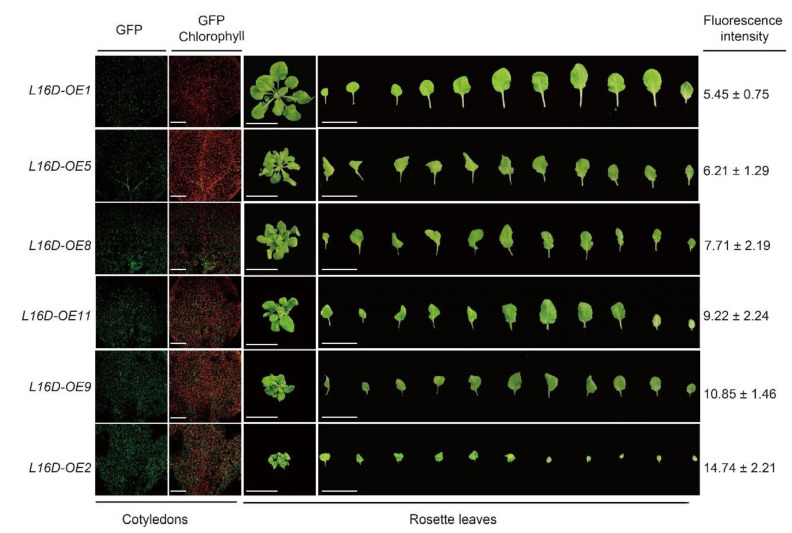
An abnormal leaf phenotype is positively correlated with the RPL16D dosage. The confocal images of five DAG cotyledons show the GFP fluorescence of the indicated lines. The phenotypes of representative plants at 25 DAG are shown. The fluorescence intensity of GFP in each line was indicated.

Additionally, varying degrees of leaf morphology among different *L16D-OE* lines prompted us to examine the potential effect of RPL16D dosage on the phenotype. GFP fluorescence intensity in *L16D-OE* lines was used as the indicator for the RPL16D accumulation level. By comparing the GFP signal intensity in 5 DAG seedlings among different lines, we confirmed that the RPL16D dosage was positively correlated with the short and curled leaf morphology (Figure 3).

### 2.2. Phylogenetic Analysis of RPL16 Genes

Arabidopsis possesses four *RPL16* genes, namely *RPL16A*–*D*. *RPL16A*, *RPL16B*, and *RPL16C* share 84.70%, 91.26%, and 85.97% of the nucleotide sequence identity of *RPL16D* (Figure S2A). The amino acid sequences of RPL16B, RPL16C, and RPL16D were the same, while the 148th threonine was converted into alanine in RPL16A (Figure S2B). We analyzed the sequences of *RPL16* genes and constructed the phylogenetic trees based on the CDS (coding DNA sequence) and protein sequences (Figure S3). The phylogenetic tree based on the CDS was more accurate because of the high amino acid sequence identity (Figure S3A). This phylogenetic tree showed that the expansion of *RPL16* genes in Arabidopsis was resulted from gene duplication events. *RPL16A* and *RPL16C* were paralogs, while *RPL16B* and *RPL16D* were paralogs (Figure S3A). The mutation among these four *RPL16* genes were mainly synonymous mutations (Figures S2 and S3). Phylogenetic trees also indicated that *RPL16s* were more similar among the proximal species (Figure S3).

### 2.3. Expression Pattern of RPL16D and Subcellular Localization of Its Product

The tissue expression patterns of *RPL16s* were first analyzed using online public data https://bar.utoronto.ca/eplant/(accessed on 11 April 2023) (Figure S4). The *RPL16B* and *RPL16D* expression levels are annotated identically on the website (Figure S4). *RPL16A* and *RPL16C* showed lower expression levels than those of *RPL16B* and *RPL16D* in all examined tissues (Figure S4). The expression of all four genes is higher in rosette leaves and roots compared to other tissues (Figure S4).

**Figure 4:**
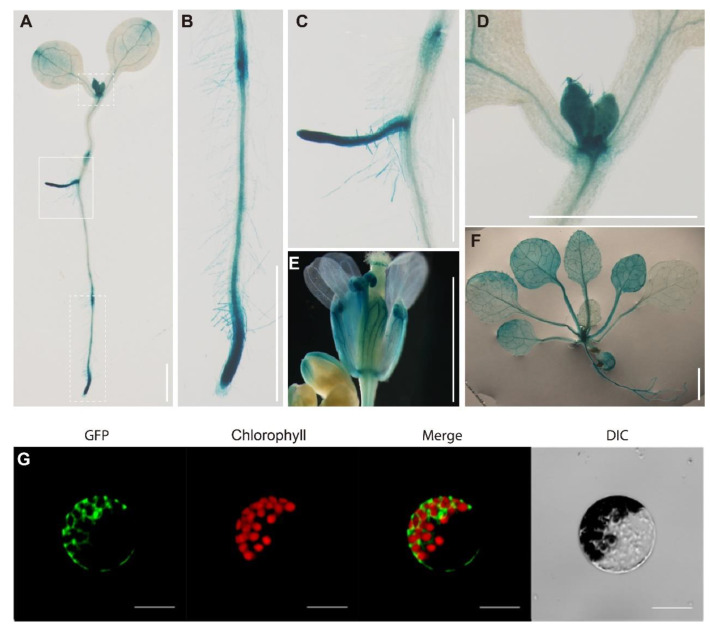
Expression pattern of *RPL16D* and subcellular localization of RPL16D. (**A**) Histochemical GUS staining in a 7 DAG seedling; (**B**–**D**) the close-up pictures of the white-dotted boxes in (**A**); (**E**,**F**) histochemical GUS staining in a flower and rosette leaves. Bars: 5 mm; and (**G**) cellular localization of the RPL16D protein. Scale bars, 10 μm.

GUS staining analysis was further carried out to examine the expression pattern of *RPL16D* in different tissues and organs. In this experiment, GUS was driven by a 2001 bp promoter of *RPL16D* in the transgenic plants. GUS signals were clearly detected in active growth regions, such as cotyledon tips, shoot apices, the primary root tip, newly emerged lateral roots, expanded rosette leaves (13 DAG), and flower buds, which was largely consistent with the online data (Figure 4A–F).

Ribosomal proteins are involved in ribosome biogenesis in the nucleus and functions as important structural components of the mature ribosome in the cytosol. To test the subcellular localization of RPL16D, we examined the presence of RPL16D-GFP fusion protein in the *L16D-OE5* leaf protoplasts. Our results indicate that RPL16D-GFP fusion proteins were mostly detected in the cytosol, where RPL16D functioned as a component of the mature ribosome in translation (Figure 4G).

### 2.4. RPL16D Might Balance Plant Growth and Immune Response

**Figure 5:**
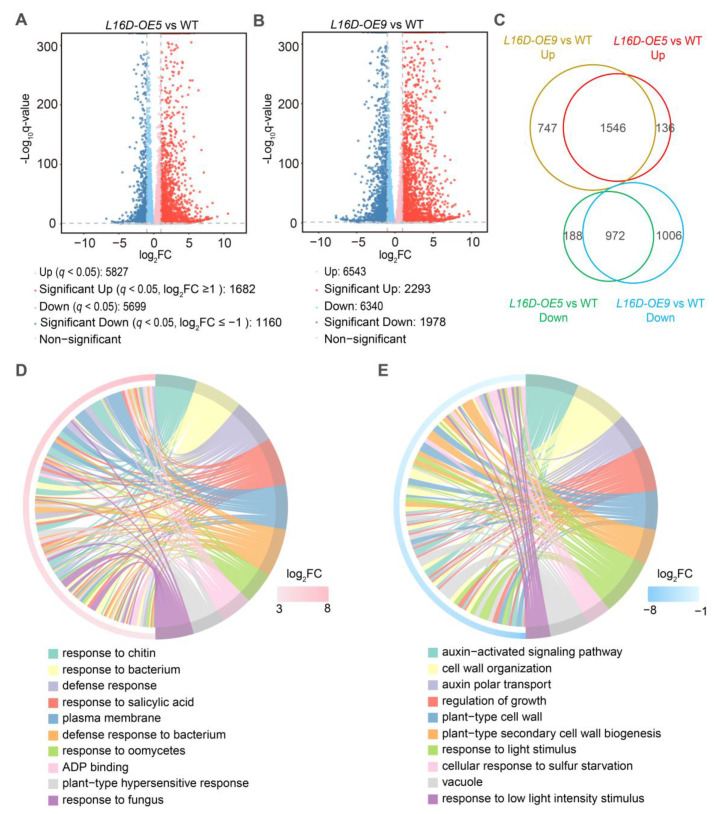
Transcriptomics analysis of WT, *L16-OE5*, and *L16-OE9*. (**A**,**B**). Volcano plots of fold changes for transcriptomic analysis. Log_2_ of fold-change and −log_10_ of q-value are present on the axis. Red, blue, and gray dots represent significantly up-, down-, and nonregulated genes (|Log_2_ (fold-change)| < 1, q-value > 0.001). (**C**). Venn diagram analyzes the differentially expressed genes (DEGs) in *L16D-OE5* and *L16D-OE9* plants. (**D**,**E**) GO functional categories of genes with up- and downregulated levels. The color in each cell indicates −log_10_ (*p*-values) of the GO enrichment according to the scale. Identification of significantly (*p*-values < 0.001) enriched GO categories.

**Figure 6:**
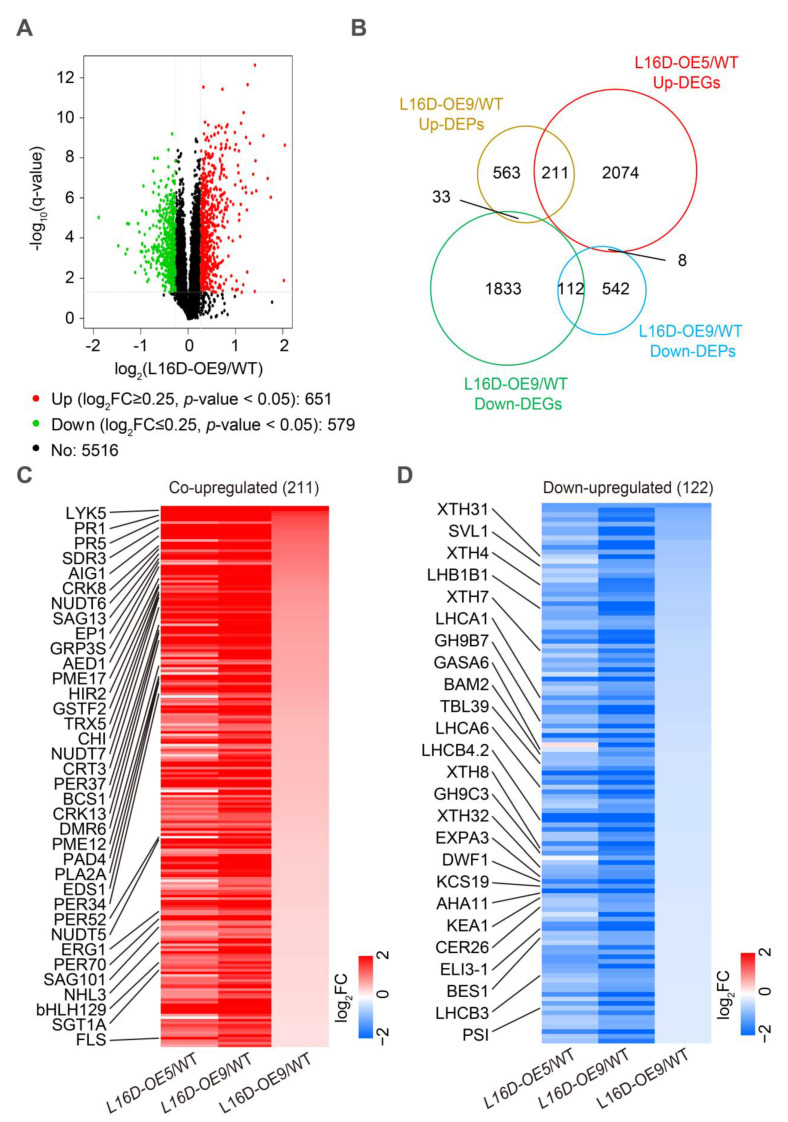
Proteomic analysis of WT, *L16-OE5*, and *L16-OE9*. (**A**). Volcano plots of fold changes for proteomic analysis. Log_2_ of fold-change and −log_10_ of q-value are present on the axis. Red, green, and black dots represent significantly up-, down-, and nonregulated proteins (**|**Log_2_ (fold-change)**|** < 1, *q*-value > 0.001). (**B**). Venn diagram analyzes the DEGs and differentially expressed proteins (DEPs) that overlapped in the transcriptomic and proteomic analyses of *L16D-OE9* plants. Co-downregulated (**C**) and co-upregulated (**D**) genes and proteins use the log_2_-transformed fold-change values. Representative proteins are labeled on the left. Red, blue, and white indicate an increase, decrease, and no difference in expression levels, respectively.

To address the molecular mechanism of leaf morphology regulation by RPL16D, we performed transcriptomic and proteomic analysis using 21 DAG rosette leaves of WT, *L16D-OE5*, and *L16D-OE9*. In the transcriptomic analyses, a total of 2842 (1182 significantly upregulated genes and 1160 significantly downregulated genes) and 3271 (2293 significantly upregulated genes and 1978 significantly downregulated genes) differentially expressed genes (DEGs) (|log_2_(fold-change)| ≥ 1 and *q*-value < 0.05) were identified in *L16D-OE5* and *L16D-OE9*, respectively (Figure 5A,B, Datasets S1 and S2). A total of 1546 co-upregulated DEGs and 972 co-downregulated DEGs were identified in *L16D-OE5* and *L16D-OE9* (Figure 5B). The Gene Ontology (GO) enrichment analysis showed that the upregulated genes related to immune response were enriched (Figure 5C), while the downregulated genes related to plant growth were enriched (Figure 5C). In proteomic analysis, 1230 (651 significantly upregulated proteins and 579 significantly downregulated proteins) differentially expressed proteins (DEPs) (|log_2_(fold-change)| ≥ 0.25 and *p*-value < 0.05) were identified in *L16D-OE9* (Figure 6A, Dataset S3). A total of 211 co-upregulated and 112 co-downregulated DEGs and DEPs were obtained from transcriptomic and proteomic analysis (Figure 6B). GO enrichment analysis showed that many DEPs related to pathogen resistance were enriched (Figure S5).

Among the co-upregulated DEGs and DEPs, we found that the accumulation of systemic acquired resistance (SAR)-related DEGs and DEPs was significantly increased, including ENHANCED DISEASE SUSCEPTIBILITY 1 (EDS1), PHYTOALEXIN DEFICIENT 4 (PAD4), PATHOGENESIS-RELATED GENEs (PR1 and 5), EP1, and ARABIDOPSIS EDS1-DEPENDENT 1 (AED1) (Figure 6C). Furthermore, the accumulation of DEGs and DEPs involved in immune response was also significantly upregulated, including LYSM-CONTAINING RECEPTOR-LIKE KINASE 5 (LYK5), CYSTEINE-RICH RECEPTOR-LIKE PROTEIN KINASE 13 (CRK13), SHORT-CHAIN DEHYDROGENASE/REDUCTASE 3a (SDR3), CALRETICULIN 3 (CRT3), PHOSPHOLIPASE A 2A (PLA2A), SALICYLIC ACID GLUCOSYLTRANSFERASE 1 HOMOLOG A (SGT1A), and NUDIX HYDROLASE HOMOLOGs (NUDT5, 6 and 7) (Figure 6C). Salicylic acid (SA) plays an important role in plant-pathogen resistance [23]. We found that the accumulation of proteins involved in the SA response was also significantly increased, including CYTOCHROME BC1 SYNTHESIS (BCS1) and GLYCINE RICH PROTEIN3S (GRP3S) (Figure 6C). Furthermore, the accumulation of some other pathogen resistance proteins was also significantly increased, including HYPERSENSITIVE INDUCED REACTION 2 (HIR2), THIOREDOXIN H-TYPE 5 (TRX5), and SENESCENCE-ASSOCIATED GENE 101 (SAG101) (Figure 6C). Among the co-downregulated DEGs and DEPs, the accumulation of sterols and BR biosynthesis protein DWARF1 (DWF1) and BR signaling proteins BRI1-EMS-SUPPRESSOR 1 (BES1) was significantly decreased (Figure 6D). Plant cell-wall biogenesis and remodeling play a critical role in leaf expansion, petiole elongation, and stem elongation [24,25,26]. Our results show that the accumulation of plant cell-wall biogenesis and remodeling-related proteins were significantly decreased in the proteomic analysis, including EXPANSIN3A (EXP3A), XYLOGLUCAN ENDOTRANSGLUCOSYLASE/HYDROLASEs (XTH4, 7, 8, and 32), GLYCOSYL HYDROLASEs (GH9B7 and 9C3), and PLC-like phosphodiesterase proteins SHAVEN 3-like 1 (SVL1) (Figure 6D). The accumulation of proteins involved in a light signaling complex was decreased, including CHLOROPHYLL A/B-BINDING PROTEINS (LHCA1, LHCA6, LHB1B1, LHCB3, and LHCB4.2), and PHOTOSYSTEM I P SUBUNIT (PSI-P) was significantly decreased (Figure 6D). In addition, we also found that several proteins involved in cell growth were significantly decreased, including GA-STIMULATED ARABIDOPSIS6 (GASA6), and BARELY ANY MERISTEM 2 (BAM2) (Figure 6D). These results reveal that *RPL16D* overexpression might affect the processes of BR biosynthesis and signaling, cell wall biosynthesis and remolding, light signaling, and cell growth (Figure 6D).

Taken together, our results indicate that the *RPL16D* overexpression inhibits the accumulation of plant growth-related proteins but induces the expression of many pathogen resistance-related genes, especially *PR1* being upregulated by approximately 78-fold in the mRNA level and by 3-fold in the protein level in *L16D-OE9* (Figure 6 as well as Datasets 2 and 3, respectively).

## 3. Discussion

RPL16 plays an essential role in peptidyl transferase activity in bacteria and yeast by associating with the small subunit, binding aminoacyl-tRNA (aa-tRNA), and organizing the architecture of a functional site in ribosomes [27,28]. In Arabidopsis, the expression of *RPL16A* and *RPL16B* in lateral root primordia (LRPs) is significantly upregulated upon auxin treatment [14]. Both our results and online data show the active expression of *RPL16D* in proliferating tissues. The independent mutation of paralogous RP genes in yeast has been reported to cause distinguishable phenotypes, suggesting that paralogous genes have distinct functions despite the high similarity of protein sequences [7,29]. Therefore, although the *RPL16* genes encode highly similar proteins, we cannot rule out the possibility that RPL16D has a specific function.

The phenotypic characterization of the *RPL16D* overexpression lines *16D-OE5* and *16D-OE9* shows that the transgenic plants display an inhibited-growth phenotype at the late vegetative growth stage (after 21 DAG) in an RPL16D protein dosage-dependent manner. It has been reported that the proper composition of membrane sterol is not only required for membrane integrity, permeability, and fluidity, but it also affects the signaling cascades involved in normal plant growth [30,31]. Our results show that the accumulation of the DWF1 protein associated with sterols and BR biogenesis is reduced. Studies of the BR-deficient and BR-insensitive mutants indicated that the dwarf stature and short rosette leaves are caused by a reduced cell size [32,33]. In addition, cell growth is also largely controlled by cell-wall extensibility [24,25,26]. Our results reveal that the accumulation of proteins for cell-wall biosynthesis and remodeling proteins (EXP3A, XTHs, GH9s, and SVL1) was downregulated. Overall, the downregulation of proteins that control plant growth in *L16D-OE5* and *L16D-OE9* is likely responsible for the abnormal leaf development.

Unlike animals, sessile plants have evolved a complex strategy to adapt to different environmental conditions and pathogen attacks. To date, little research has focused on the role of RP and ribosome heterogeneity in plant pathogen resistance. Here, we found that many pathogen resistance genes and proteins were upregulated in our transcriptomic and proteomic results. The constitutive overexpression of *EDS1* together with *PAD4* was reported to cause an autoimmunity phenotype (short rosette leaves), resembling the phenotypes of *L16D-OEs*, whereas the overexpression of *EDS1* or *PAD4* alone did not cause this phenotype [34]. EDS1-PAD4 works in parallel with SA and MAPK, which confers a flexible pathogen resistance system [34,35,36]. However, it is not known whether autoimmunity directly contributes to the inhibited growth in *L16D-OEs*. Here, upregulated *PR1* and *PR5* in *L16D-OEs* might contribute to the enhanced pathogen resistance. Taken together, we found that the overexpression of *L16D* leads to inhibited growth and enhanced pathogen resistance.

Transgenic plants with a moderately increased RPL16D dosage (such as *L16D-OE5*) display weak growth inhibition but still have enhanced pathogen resistance. Furthermore, a previous study reports that plants overexpressing *PR1*, *PR2*, and *PR5* show drought-tolerance phenotypes [37]. Therefore, our results suggest that increasing the RPL16D dosage might have implications for the genetic improvement of crops.

## 4. Materials and Methods

### 4.1. Plant Materials and Growth Conditions

Arabidopsis accession Columbia (Col-0) was used as the wild-type (WT) background in this study. Seeds were surface-sterilized using H_2_O_2_ (10%) and plated on solid 1/2 Murashige and Skoog (MS) medium containing 1% sucrose (pH 5.7). After a 3 d stratification, plates were transferred to the growth room (21 °C, 14 h light/10 h dark cycles).

### 4.2. Construction of Transgenic Plants

To generate an *RPL16D* overexpression construct (with or without a GFP tag), the full-length coding sequences of *RPL16D* (*At5g45775*) were amplified with or without a stop codon using specific primers. The latter was fused with a C-terminal GFP-tag. *RPL16D* and *RPL16D-GFP* fusion sequences were cloned into *pCAMBIA2300* driven by the cauliflower mosaic virus (CaMV) *35S* promoter. The 2001 bp promoter of *RPL16D* was amplified and cloned into *pBI121* to generate a *pRPL16D*:*GUS* construct. The above constructs were transformed into WT Arabidopsis using the *Agrobacterium tumefaciens* (GV3101)-mediated floral dip method to transform Arabidopsis plants. Transgenic plants were screened using kanamycin. All primers used in this study were listed in Table S1.

### 4.3. Detection of GFP Fluorescence Signals

GFP signals in five DAG cotyledons and leaf protoplasts were detected using confocal laser scanning microscope (488 nm excitation, OLYMPUS FV1200, Olympus Corp., Tokyo, Japan). Protoplasts were isolated from *L16D-OEs*. The protoplast isolation was previously described [38]. At least 15 cotyledons (GFP intensity) and 10 protoplasts (protein subcellular location) were used for confocal microscopy.

### 4.4. Histochemical GUS Staining

Seedlings and rosette leaves were stained for GUS activity by incubation in the GUS staining buffer (0.05 M NaPO_4_ buffer (pH 7.0), 5mM K_3_Fe(CN)_6_, 5mM K_4_ Fe(CN)_6_ and 2 mM X-glucuronide) overnight at 37 °C [39]. The material was passed through an ethanol series (70%, 50%, and 20%) and finally stored in 50% *v*/*v* glycerol for photography.

### 4.5. Transcriptomic Analysis

Total RNA was extracted from 20 DAG rosette leaves from three independent biological replicates using RNAiso (Takara). DNase I (Takara) was used to remove genomic DNA according to the manufacturer’s protocol as described previously [40]. The RNA quality was analyzed using the Agilent 2100 Bio analyzer (Agilent RNA 6000 Nano Kit, Agilent Technologies, Santa Clara, CA, USA) and NanoDrop. The mRNA was enriched using the Oligo dT Selection method and cleaved into short fragments. Fragmented mRNAs were used to obtain double-stranded cDNA (dscDNA) by the N6 random primer. The synthesized dscDNA was subjected to end-repair and was then 3′ adenylated. Adaptors were ligated to the ends of these 3′ adenylated dscDNA fragments. The ligation products were purified, and PCR amplification was performed to enrich the purified cDNA template. The PCR product was denatured by heat and the single-strand DNA was cyclized by a splint oligo and DNA ligase to construct the cDNA library. Three biological replicates of each sample were used for RNA-Seq using the BGISEQ-500 platform by BGI. The low-quality reads, reads with adaptors, and reads with unknown bases (N bases more than 5%) were filtered to obtain clean reads using the SOAPnuke software version 2.0. All clean reads were aligned with the Arabidopsis TAIR10 reference genome using Bowtie2 (http://bowtie-bio.sourceforge.net/Bowtie2/index.shtml). The gene expression level was calculated with RSEM (http://deweylab.biostat.wisc.edu/RSEM 13 March 2023) and normalized using the fragments per kilobase of transcript per million mapped reads (FPKM) method. Differentially expressed genes (DEGs) were identified using DEGseq software version 1.0 (fold change ≥ 2 and *p*-value ≤ 0.001) and used the Volcano Plot to show the summary of DEGs.

### 4.6. Proteomic Analysis

Rosette leaves of WT and *L16D-OE9* (20 DAG) from three independent biological replicates were finely ground in liquid nitrogen for protein extraction. Raw MS/MS data were analyzed using ProteoWizard Tool msConvert (version 3.0.1), and then peptides were identified using TAIR10_1015 (40,709 sequences). At least one unique peptide was necessary for an identified protein. Differential accumulation ratios of proteins were analyzed by the automated software IQuant (version 2.2.1). To calculate the differential accumulation ratios, all identified spectra from a protein were used to obtain an average protein ratio relative to the control label (i.e., fold change). We used *p* < 0.05 and the fold change >1.2-fold or <0.83-fold as the thresholds to judge the significantly differentially expressed proteins (DEPs).

### 4.7. Gene Ontology Analysis

GO annotation was classified into biological process ontology using Blast2GO and the significantly enriched GO terms were identified using a hypergeometric test under the standard of the *p*-value ≤ 0.001. The pictures were made as previously described [40,41].

### 4.8. Accession Number

The accession numbers of the genes used in this article are as follows: *RPL16A* (*AT2G42740*), *RPL16B* (*AT4G18730*), *RPL16C* (*AT3G58700*), *RPL16D* (*AT5G45775*), *EDS1* (*AT3G48090*), *PAD4* (*AT3G52430*), and *PR1* (*AT2G14610*).

## Data Availability

Not applicable.

## Supplementary Materials

The following supporting information can be downloaded at: https://www.mdpi.com/article/10.3390/ijms24119479/s1.
