# Supplementary material for: Overexpressing Ribosomal Protein L16D Affects Leaf Development but Confers Pathogen Resistance in Arabidopsis
Source: Int J Mol Sci. 2023 May 30;24(11):9479. doi: 10.3390/ijms24119479 (PMC10253392; doi:10.3390/ijms24119479)
Supplement: Supplementary file 1 [file ijms-24-09479-s001.zip › Supporting information.pdf]

Overexpressing Ribosomal Protein L16D Affects Leaf Development but Confers Pathogen Resistance in Arabidopsis

Ke Li[1#], Zhenwei Yan[3#], Qian Mu[1], Qingtian Zhang[1], Huiping Liu[1], Fengxia Wang[1], Ao Li[1], Tingting Ding[1, 2], Hongjun Zhao[1], Pengfei Wang [1, 2*]

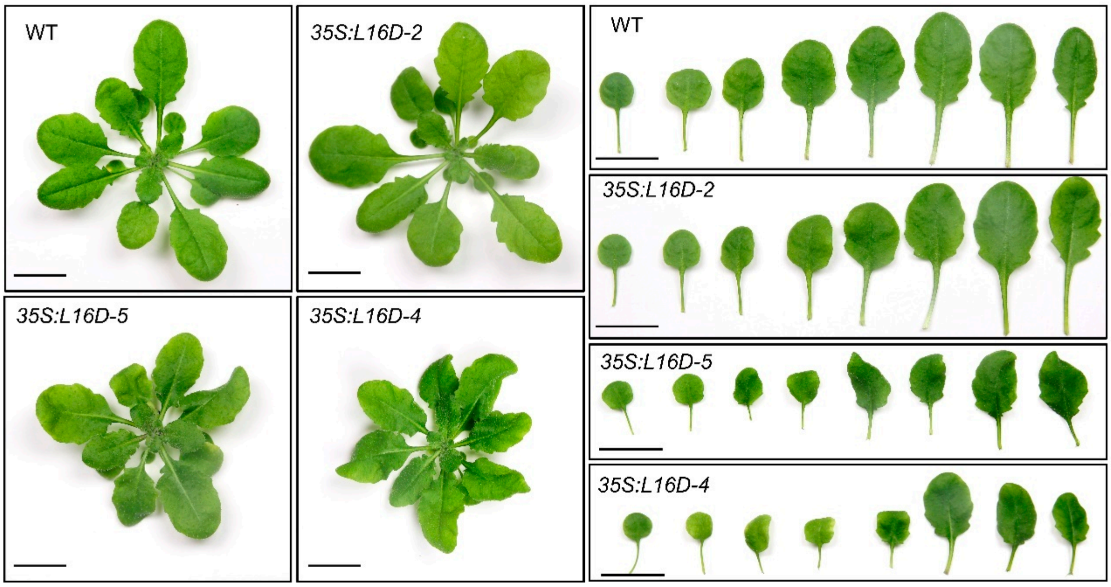

Figure s1. The phenotype of plants overexpressing *RPL16D* without a tag. 25 DAG plants (scale bars, 1.5 cm) and fully expanded eight rosette leaves (scale bars, 8 mm).

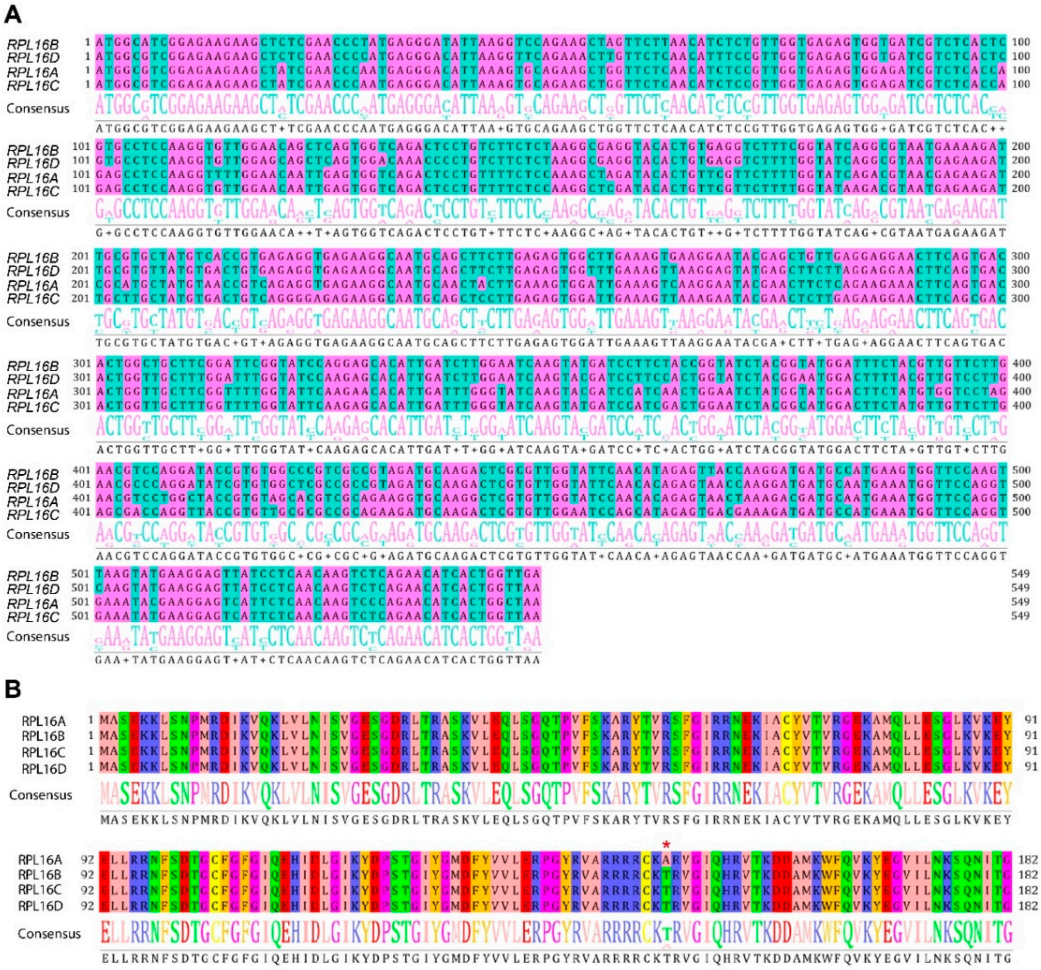

Figure s2. Multiple sequence alignment of RPL16. A. *RPL16* family CDS sequences alignment. B. RPL16 family protein sequences alignment. A red asterisk indicates amino polymorphism in RPL16A.

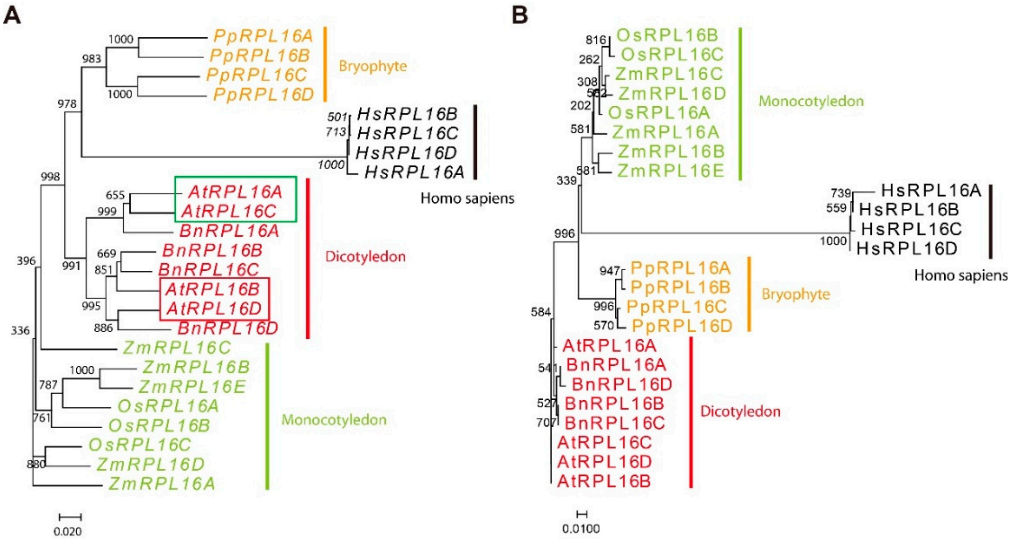

Figure s3. Phylogenetic analysis of *RPL16D*. (a-b) Phylogenetic trees based on the

CDS (Coding DNA Sequence) sequences (a) and proteins sequences (b). *A. thaliana*, (*AtRPL16*), *B. napus* (*BnRPL16*), *H. sapiens* (*HsRPL16*), *O. sativa* (*OsRPL16*), *P. patens* (*PpRPL16*), *Z. mays* (*ZmRPL16*).

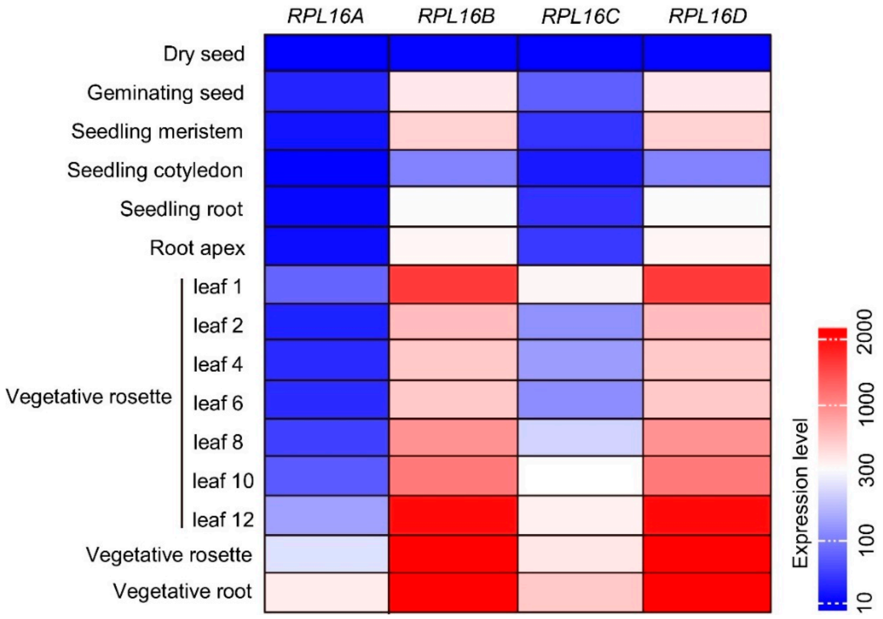

Figure s4. Expression pattern of *RPL16D* in public data. (ePlant: https://bar.utoronto.ca/eplant/).

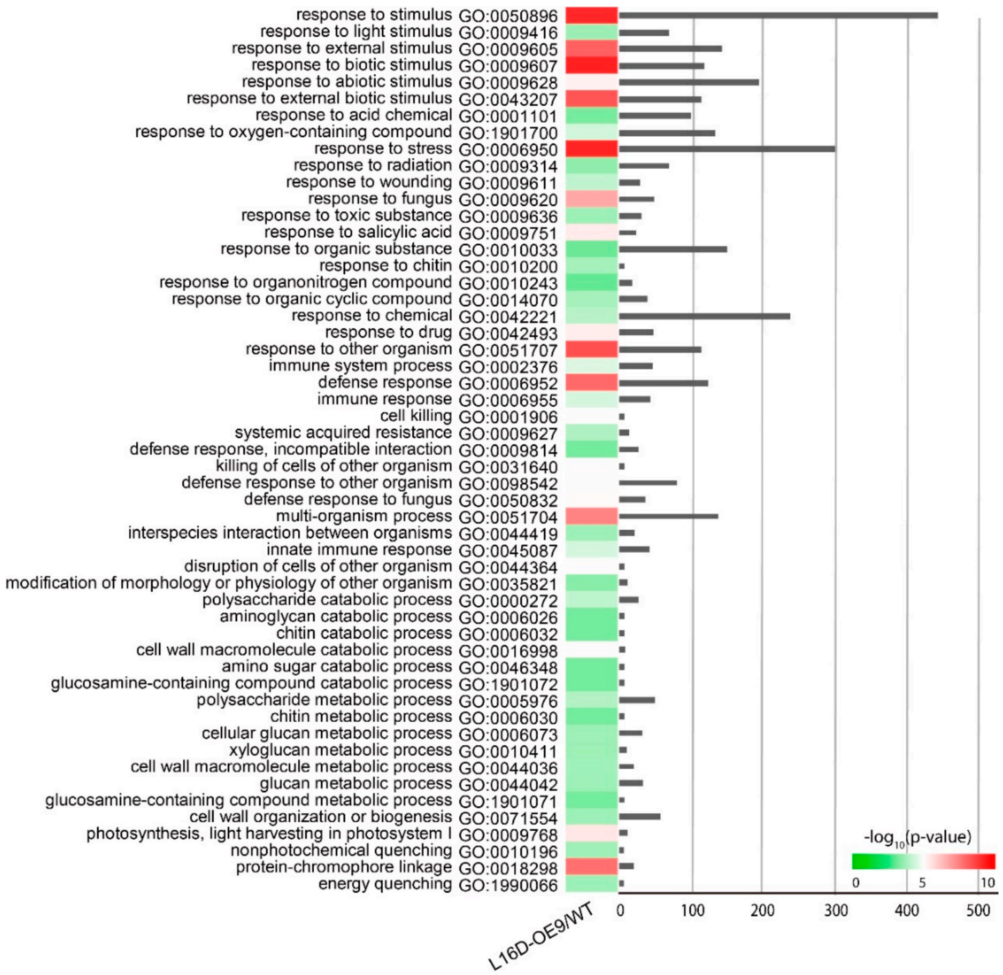

Figure s5. GO analysis for proteomics analysis. GO functional categories of proteins with up- and down-regulated levels. The color in each cell indicates -log10 (*P*-values) of the GO enrichment according to the scale. Identification of significantly (*P*-values < 0.001) enriched GO categories.

Table S1 Primers used in the study.

Primer name	Sequence (5'-3')
RPL16D-GUS F	cggGGTACCTCCCAAGAGCTTTGCTCAGATGCT
RPL16D-GUS R	cgcGGATCCTTTAAGGAGATCAATGTCGAGCTA
RPL16D-OE F	CGGGGTACCATGGCGTCGGAGAAGAAGCTCTCG
RPL16D-OE R	CGCGGATCCTTAACCAGTGATGTTCTGAGACTT
RPL16D-GFP F	CGGGGTACCATGGCGTCGGAGAAGAAGCTCTCG
RPL16D-GFP R	CGCGGATCCTTAACCAGTGATGTTCTGAGACTT
